# Research on the protection of athletes from injury by flexible conjugated materials in sports events

**DOI:** 10.3389/fchem.2023.1313139

**Published:** 2023-11-29

**Authors:** Jian Liu, Tingting Ren

**Affiliations:** ^1^ Physical Education Institute, Shangqiu University, Shangqiu, Henan, China; ^2^ Department of Public Education, Xinjiang Vocational and Technical College Construction, Urumqi, Xinjiang, China

**Keywords:** nanomaterials, flexible conjugated materials, athlete event, and sports injury, protection of athletes

## Abstract

Sports are essential to everyone’s health because they assist athletes to establish physical and mental balance by strengthening muscles and ligaments. High-intensity training and low-quality equipment for sports tend to cause a wide range of injuries to the athlete. Higher education graduates’ regular education and lives are disrupted, either directly or indirectly, by sports injuries. Therefore, understanding the prevalence and root causes of college athletes’ injuries is crucial for enhancing student athletes’ performance and fostering healthy development. The ever-changing nature of injuries associated with sports and the patchy availability of rehabilitation facilities across India cause alarm. Inaccurately identifying players’ physical indications, uncomfortable clothing, and dissatisfaction with sports equipment are among the issues that can arise. The study investigates the potential of nanoparticles combined with sports flexible conjugate materials for injury prevention in athletes. The article proposed nanotechnology combined with flexible conjugated materials in sports events (Nano-FCM-SE) in sports training, explores the possibility of conjugated materials in enhancing the training effects of athletes, monitoring the status of sports, and bettering equipment. Sports equipment can help keep athletes safe by incorporating nanotechnology and flexible conjugated materials with superior optical, electrical, and other capabilities. Convenience, waterproof materials, flexibility, lightweight, aesthetics, breathability, and durability are evaluated for use in Nano-conjugated sports equipment materials. Evidence suggests that using flexible conjugated materials in athletic training can improve athlete performance and help the overall development of sports. The proposed method yields less negative results than MSI-TENG, TCM-MS, and RANSAC. The proposed damage severity model performs poorly relative to competitors (0.2). Compared to conventional models, the given models are effective on equipment. The sports injury protection system reported in this research has 5.17 percentage points greater detection efficiency than the current state of the art. Hierarchical strategies have the best RMSE for athlete safety. The findings of such methodologies in athlete safety on Nano conjugate materials and sports biology on sporting events and equipment underline the importance of precise data for athlete safety and performance.

## 1 Introduction

All educational institutions emphasize the importance of regular sports and encourage their students to participate in sports and other recreational activities. However, sports have a significant injury burden; studies evaluating injury protection strategies are needed in all activities and ages (Gao et al., 2022). Sports are the leading source of injury among young people. Recovering from sports injuries is a highly specialized field in the present era, which naturally necessitates the collaboration of sports physiotherapists and physicians (Willwacher et al., 2023). Participation rates are higher among children and teenagers. One-third of young people annually seek treatment for sports-related injuries, and statistics indicate that 30% of pupils will be unable to attend a minimum of a single day of school owing to sports injuries. Injuries sustained while participating in sports account for at least 1 day per year of lost productivity for individuals (Miller et al., 2021). Injury rates among adults are higher than among children and teenagers because of sports. In addition, the economic costs associated with sports injuries are substantial (Zhou and Chu, 2022). Evidence-driven injury mitigation strategies are needed to lower the risk of injury in young people and throughout their lives, as shown by the high incidence of injuries and associated expenditures. Injury is an inevitable by-product of sports competition, play, and development (Rodriguez-Santiago et al., 2019). Athletes can raise their performance levels through training. Athletes’ skills can be evaluated in part through competition. However, sports injuries brought on by inappropriate, overloaded practice or erroneous professional motions might disrupt regular workouts and enhance competition outcomes (Javed et al., 2022). Therefore, players must be familiar with typical sports injuries, preventative measures, and recovery methods.

Injury prevention in sports was suggested in the paper through the use of Nano-FCM-SE items, which have advanced sensor technology, electrical, and other qualities. The use of nanomaterials in sporting equipment has led to gains in speed, agility, durability, and portability. Research into nano-conjugated materials for sports equipment aims to improve performance and reduce the risk of injury for athletes. It has been established that incorporating flexible conjugated materials into athletic training enhances athlete performance and helps the sports sector expand.

It is concerning that the nature of injuries associated with sports is evolving and that rehab facilities are lacking in many parts of India. As the athletics industry becomes increasingly competitive and economically rewarding for athletes, many aspire to the highest levels of professionalism (Younas et al., 2021). Because of this, athletes are now under a more significant psychological and physiological strain than ever before, subjected to more rigorous training and practice schedules, and at greater risk of injury. In today’s competitive sports, players who sustain injuries typically face demands from the individual and the team’s management to return to action as soon as feasible (Wang, 2022). Because of the intense competition, athletes risk being left off the roster and, as a result, face more significant incentives to get back into action. Therefore, sports injuries require more attention to a highly structured and sports-specific approach to rehabilitation, which should prepare the athlete and the injured tissue for the subsequent demands placed on them at the pinnacle of the sport (Ansari, 2022). The model is mainly constructed using nanogenerators built from nanotextiles. The athlete’s motion produces energy, and the nanosensor made of conjugate materials records the specifics of that motion. Information gathered to monitor athlete safety is presented in this article via a mobile application or system.

Even though cricket remains the most famous sport in India, the country is developing a strong sports culture, as evidenced by the rising popularity of the newly established sport. Athletes in these competitions face a considerable risk of exhaustion and injury because of the games’ rapid pace and short duration (Cai et al., 2023). International research consistently highlights the link between the physical demands of sport and the associated dangers. India lags in injury prevention and control compared to more advanced nations due to a lack of studies and research on established programs targeting Indian athletes (Tan et al., 2023). The study discusses conjugate materials, which are elements with improved efficiency and more usefulness due to advances in technology and science and find widespread application in the athletic field (Cao et al., 2019; Lv et al., 2022). Sports and fitness equipment have benefited from its widespread adoption (Rao et al., 2023). The nanomaterial applications highlight the significance of using flexible conjugate materials in sports, as they improve player safety and comfort (Han et al., 2022). Since its early days, the tribo effect has found several applications, including harvesting energy and a wide range of self-driven mechanical sensors. Tribo nanotechnology has evolved over the past few years, with flexible textile materials gradually becoming the primary material. It has potential in clothes, greatly simplifying charging people’s many wearable electronics. There have been preliminary attempts to integrate this energy harvesting into human apparel. The produced tribo nanogenerator can be placed to the insole or attached to the sensor to supply electrical energy to some micro devices; furthermore, several textile materials can be used as the primary friction-reducing component of the device. Insufficient utilization rates in athletic training, inadequate and sophisticated study and improvement content, and high manufacturing expenses of firm supplies and supplies made from it are just a few of the issues with using conventional supplies in sports education that have arisen as activities training has progressed (Chen-Yu et al., 2022). This paper takes nanomaterials used in equipment used in sports events, such as baseball bats, hockey clubs, volleyball and handball racquets, golf clubs, and fly-fishing rods combined with conjugation as the central topic of discussion. It clarifies the current application of conjugation materials in athletic competitions to address these issues. Nanomaterials have become the subject of intense study and development over the last decade, finding use in areas as diverse as catalysis, medicine, sensing, and biology. The performance, adaptability, durability, and portability of sports equipment made with nanomaterials are all improved. In addition to keeping them safe, the athlete’s equipment can significantly impact their performance (Li et al., 2022). Nanomaterials, with their size-dependent qualities, significantly affect many areas of use, including building materials, consumer goods, and medicine. Using Nanoscale conjugated materials in athletic training aims to advance these substances’ more systematic and complete utilization in this context.

The impact of various materials on athletic training is varied. When used in sports training equipment and equipment, conjugate materials can increase the safety, comfort, and sports effectiveness in sports, which is of significant relevance.

However, there are still issues with the waterproofing and transpiration of apparel, and there are inaccuracies in the equipment for tracking athletes’ physical indicators, even though using various fabrics in athletics clothing and equipment has resulted in many benefits. The features of conjugated materials, such as monitoring and waterproofing, provide efficient solutions to these issues.

The purpose of this analysis is to provide a synthesis of previous studies that have looked at the quality and security of sports around the globe. This article combines the current status of development with a review of public sports facilities, looking at their safety and the quality of their services. At its core, it is an effort to provide technical assistance for the growth of public benefit sports by establishing a standardized framework for managing and delivering public sports. The primary goal of sports is to fulfill people’s needs for access to and enjoyment of organized physical activity. Self-contained venues should be the gold standard for public sports. Various arenas, buildings, permanent facilities, etc., are part of the sports infrastructure used for games, practices, classes, and general community health and wellness.

The main contributions of the article include

1. The article proposed using Nano-FCM-SE sports items with superior sensing technology, electrical, and other properties for injury prevention.
2. Nanomaterials in sports items offer improved performance, flexibility, durability, and lightweight. Nano-conjugated sports equipment materials are studied to enhance athlete performance and prevent injury.
3. The use of flexible conjugated materials in athletic training has been shown to increase performance in athletes and contribute to the growth of the sports industry.

The remainder of the project is then structured as follows: The varied perspectives of researchers on athletes’ effects on injuries are discussed in Section 2. The impact of nanoparticles is discussed in Section 3, Section 4 discusses flexible conjugated materials and result analysis, and Section 5 concludes the paper.

## 2 Literature survey

Ramos and Almeida (2023) examine the international standard ISO/TS 12901-2:2014, highlighted because it provides a control banding technique to reduce occupational exposure to nano-objects and their mixtures and agglomeration larger than 100 nm. The article includes a case study of a textile coating company that uses two nanomaterial-based pharmaceutical finishes. Workers interacting with nanoparticles were the focus of a risk assessment. The dangers were reduced through control banding, the prescribed measures of adequate ventilation, and the use of protective equipment. The information offered is still limited regarding the particular hazards and concerns caused by nanoparticles, even though the safety warnings are a critical source of knowledge on how to approach and care for items incorporating nanomaterials.

Zhu et al. (2023) created a Metaverse sports interactive system using triboelectric nanogenerators (MSI-TENG) for real-time communication and collaboration between humans, robots, and the web. A self-powered anaerobic power meter (APM), wireless transmission module, tailored data analysis using the industry-standard Wingate anaerobic test (WAnT) technique, and an AR application make up this Metaverse sport interactive system. WAnT-measured power (PP), mean power (MP), and fatigue index describe explosive strength, speed endurance, and endurance. Machine learning makes the professional WAnT a trustworthy predictor of anaerobic capacity and a standard for athlete selection. This study proposes a novel strategy for building the Metaverse, including online tournaments, physical training, and participant selection.

Fan et al. (2022) analyze bone and cartilage, which are tendons, connective tissue, and skeleton muscles, which comprise the conjugated Materials of the human body’s musculoskeletal (MS) system. New therapeutic conjugated Materials approaches for treating MS problems (TCM-MS) and related disorders have benefited dramatically from tissue engineering. Natural polymers have advantageous features that facilitate optimal cell contact and desirable biological function compared to other biomaterials utilized for tissue engineering. This article briefly overviews current developments in musculoskeletal tissue engineering using scaffolds derived from natural sources.

Alheib et al. (2022) offer a hydrogel composed of Gellan Gum (GG) that undergoes *in situ* cross-linking and is then combined with the skeletal muscle-inspired laminin-derived peptide RKRLQVQLSIRTC (Q) and encapsulated SMCs. C2C12-laden hydrogels injected into animals with chemically induced muscle injury enhanced myogenesis. C2C12-treated tissues had numerous myoblasts (-SA+ and MYH7+), whether the cells were free-floating in the hydrogel or encapsulated. Eight weeks following treatment with C2C12-loaded hydrogels, -SA protein levels increased significantly, and 4 weeks later, MHC protein levels increased in all experimental groups compared to the SHAM. Also identified were neovascularization and neoinnervation defects. Overall, C2C12 hydrogels show potential for skeletal muscle regeneration.

He et al. (2022) explore the design of molecules with side chains that can affect the functionality and characteristics of organic electrochemical transistors (OECTs). The article provides a brief overview of the present state of knowledge about OECT performance, emphasizing the understanding of ionic-electronic coupling, which helps illuminate the significance of side-chain growth in OMIECs. More in-depth studies into the critical impact side-chain engineering plays on the resulting OMIEC properties will pave the way for the development of side-chain alternatives, which will lead to additional improvements in the field of OECT channel materials as the area continues to evolve.

Hassabo et al. (2023) developed nanotechnology to improve engineering, industrial, technical, and medicinal textiles. Nanoparticles (NPs) and sensor integration in textile nanotechnology have increased cleaning, UV protection, flame opposition, and antimicrobial capabilities. Its adaptable sensing, robotics, electrical conductivity, flexibility, and comfort make it useful in medical treatment, athletics, advanced technology, medications, aerospace, military use, automobile manufacturing, food and agriculture, and more. Metallic fibers with copper and silver nanoparticles (NPs) are antiviral and antibacterial. This research examines nanotechnology in sports flooring, clothing, and footwear. Sports have gained essential elements. Nanotechnology-based apparel is also intensively studied. Current predictions indicate that nanotechnology will soon penetrate all sporting wear sectors.

Zhang (2022a) introduce MRDM image data of tendon injury healing acquired using medical image analysis technology, and the RANSAC algorithm is used to screen the useless image data based on the theory of tendon injury healing. The use of biomaterials is found to be beneficial in encouraging the stable healing of tendons by examining filtered MRDM image data. Several routinely employed biomaterials for tendon injury and adhesion repair were assessed for their actual effect using a multilevel model. Sodium hyaluronate was found to be the most effective treatment for tendon injuries.

Li (2022) provided that the extent of myocardial injury is studied with an intelligent optimization method. Comparing pre- and post-injury structural data can identify myocardial damage. Define the measurements and numerical model output as an objective function of structural parameters, then constantly optimize the objective process to match observed values. The objective function and three machine learning-based optimization techniques simulate damage identification for optimal computational outcomes. Computing studies show that objective function and machine learning algorithms may quickly and accurately identify myocardial injury.

Hjort et al. (2023) analyses the SINTEC Finalized Training “Smart Bioelectronic and Wearable Systems,” which aims to showcase European research on ultra-flexible, elastic, soft, and symmetrical technologies, as well as provide a forum for researchers and industry professionals to network to identify potential future markets for extensible electronic devices.

However, due to the high refractive index, inorganic filters have a whitening impact on sunscreen compositions, reducing their aesthetic appeal. Nanotechnology (Chouhan and Butola, 2023) is one of many methods explored to address this issue. Sunscreen lotions and UV-protective coatings for buildings, vehicles, and clothing all aim to shield the wearer from the Sun’s harmful ultraviolet (UV) rays. The formulations of sunscreens have been improved to be more photostable and provide protection from a broader range of UV rays. Researchers have been trying to find ways to enhance UV protection by studying the use of solid lipid nanoparticles, freshly investigated organic compounds, inorganic components, and antioxidants.

A review of the available literature shows that substantial progress has been made toward protecting athletes from harm. Nonetheless, it draws attention to several research gaps and suggests avenues for future inquiry and development. Enhancing their accuracy, interpretability, and generalizability is essential to assure the models’ practical applicability in sports injury. In light of current developments in intelligent computing, new methods have emerged to comprehend and prescribe sports injury protection using prediction models. These methods include machine learning, the RANSAC algorithm, Nanoparticles (NPs), and sensor integration.

## 3 System methodology

### 3.1 Structure of nano-FCM-SE

**FIGURE 1:**
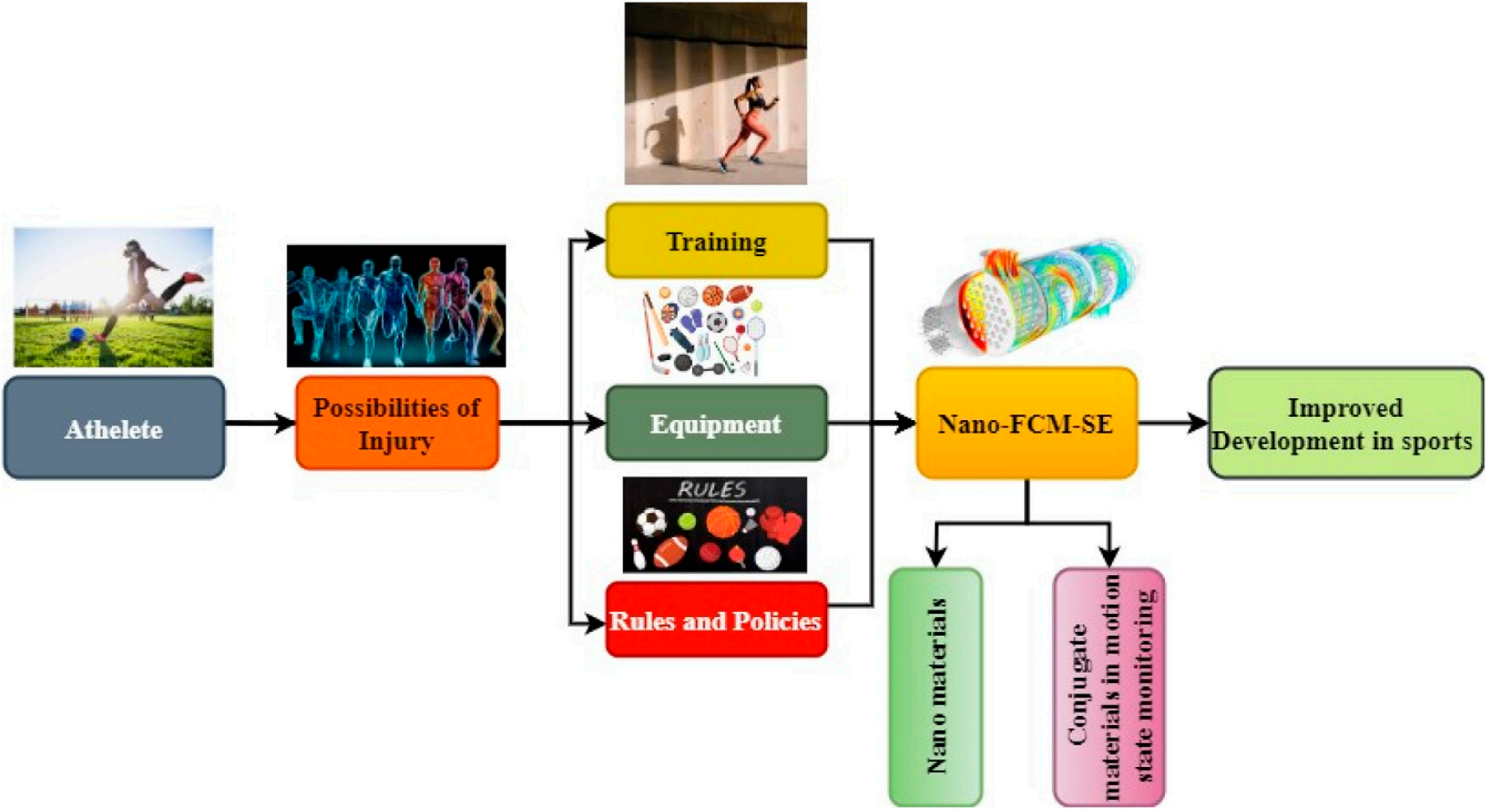
Nano scale Architecture-FCM-SE.

The goal of sports injury prevention is to reduce the number of injuries, the severity of those injuries, and the severity and breadth of their effects. Muscular exercise therapies target athlete risk variables such as strength, resilience, and stability. Rule changes and equipment initiatives tackle environmental risks (Robert, 2021; Zhang Nian, 2022). Students who include regular physical activity in their schedules report improved academic performance. Playing sports is a great way to maintain their health and focus on academics. Collaboration, managing multiple tasks, and awareness of circumstances are all transferable qualities that can help students succeed in the classroom. Figure 1 illustrates the Nano-FCM-SE, in which activities like cross-country skiing rely on continuously tracking athletes’ physiological data, technical personnel movement, and ambient information throughout training and concurrence processes to guarantee athletes’ safety and standardized technology and boost comparable outcomes. The proposed methodology mainly discusses the prevention of injury of athletes in sports events during the

• Nanotechnology-based sports equipment with flexible conjugated materials
• Nanoscale -FCM-SE in athlete event
• Rules and policies to prevent injury

Athletes must be careful during training sessions because that’s when most injuries happen. Injury prevention measures are in place during sports practice, including safety gear, protocols, and regulations. Traditional sensor methods are fragile and inflexible, limiting athletes’ range of motion and producing erroneous tracking signals as they shift and separate from the body. Outfitting athletes with adaptable hybrid electronics-based monitoring systems can help solve these issues. Conventional materials used in sports training have some problems as sports training evolves, including a low rate of utilization in sports training, a lack of advanced technological content, and a high production cost for sports education devices and apparatus made from it.

Over the past two decades, nanoscale monitoring and tracking technology advancements have been made. Commonly utilized nanosensors with thin film or substance structures are susceptible to stretching distortions and changes in organic vibration rates brought on by temperature changes, which can significantly impact the precision of sensing and detection.

The proposed Nano-FCM-SE utilizes flexible conjugated materials, which have improved productivity and more substantial efficacy due to scientific and technological progress finding applications in every sphere of human activity. It has also seen extensive application in sports practice gear. Nanotechnology combined conjugated materials on training, sports events, and equipment are essential for athletes because they can increase participant safety and comfort.

In Figure 1, the suggested Nano-FCM-SE uses flexible conjugated materials, which have increased productivity and efficacy due to scientific and technical progress finding applications in all human endeavors. It is also used in sports practice gear. It has been claimed that innovative materials will be produced through the arrangement and combination of substances on minimal dimensions, creating nanomaterials, which are components with Nanoscale structure and can be further subdivided into zero-dimensional nanotechnology and narrow-minded nanomaterials. As the diameter of a Nanocrystal particle decreases, the proportion of surface atoms to total atoms increases substantially. The formal merging of theoretical research and current scientific and technological practice did not occur until the last years of the 20th century when the United States hosted the inaugural global symposium on Nanoscience and technology. When a material’s dimensions are on the Nanoscale, the number of atoms on its surface increases considerably, far beyond the number of particles on the surface of typical materials, and the material’s chemical activity increases dramatically. These exceptional features make possible the vast array of uses for nanomaterials. As both theory and practice continue to advance, new nanostructures that cannot be classified as systems are emerging. Active sportswear’s convenience and athletic benefits are strongly related to its performance attributes.

Sensors for sensing concentrations of ions, humanoid physical sensors, synthetic transmembrane sensors, and others will all be discussed in this section, all of which use self-sustaining sensors.

The precision of sensing and detecting can be impacted by temperature fluctuations, particularly for frequently used nanosensors with thin layers or substrate structures, due to twisting distortions and variations in natural vibration frequencies.

Recovering athletic movement is an area where conjugate materials shine. This paper aims to add to the existing body of literature on the use of conjugated materials in post-game rehabilitation. In this article, sports-related athlete tiredness or injury among students at a nearby institution of physical education serves as the experimental object.

#### 3.1.1 Nanotechnology-based sports equipment with flexible conjugated materials

Over the last two decades, nanotechnology sensing and conjugate material technology advancements have been made. Surface effects are crucial to the dynamic and static characteristics of Nanoscale devices because of the large surface-to-volume ratio of these devices. The precision of detection and recognition can be impacted by changes in temperature, especially for frequently used Nanosensors with fragile films or substrate buildings, due to folding distortions and variations in natural vibration frequency. The converter’s high-sensitivity Nanosensors are often hetero-material stacked sensors, which consist of two different types of materials. The most significant factor is temperature, which factors like exposure to light, variations in the surrounding temperature, the piezoelectric effect, and molecule adsorption can alter. In the static analysis section, the deformation process of the statically indeterminate sensor is explored in depth to derive the deformation radius hypothetically and the vibrating velocity of the nanosensor beneath the influence. As a result, an analytical formula for the bending deformation’s curvature can be derived. Vibration analysis should consider the impact of the material’s temperature dependence on the vibration frequency.

**FIGURE 2:**
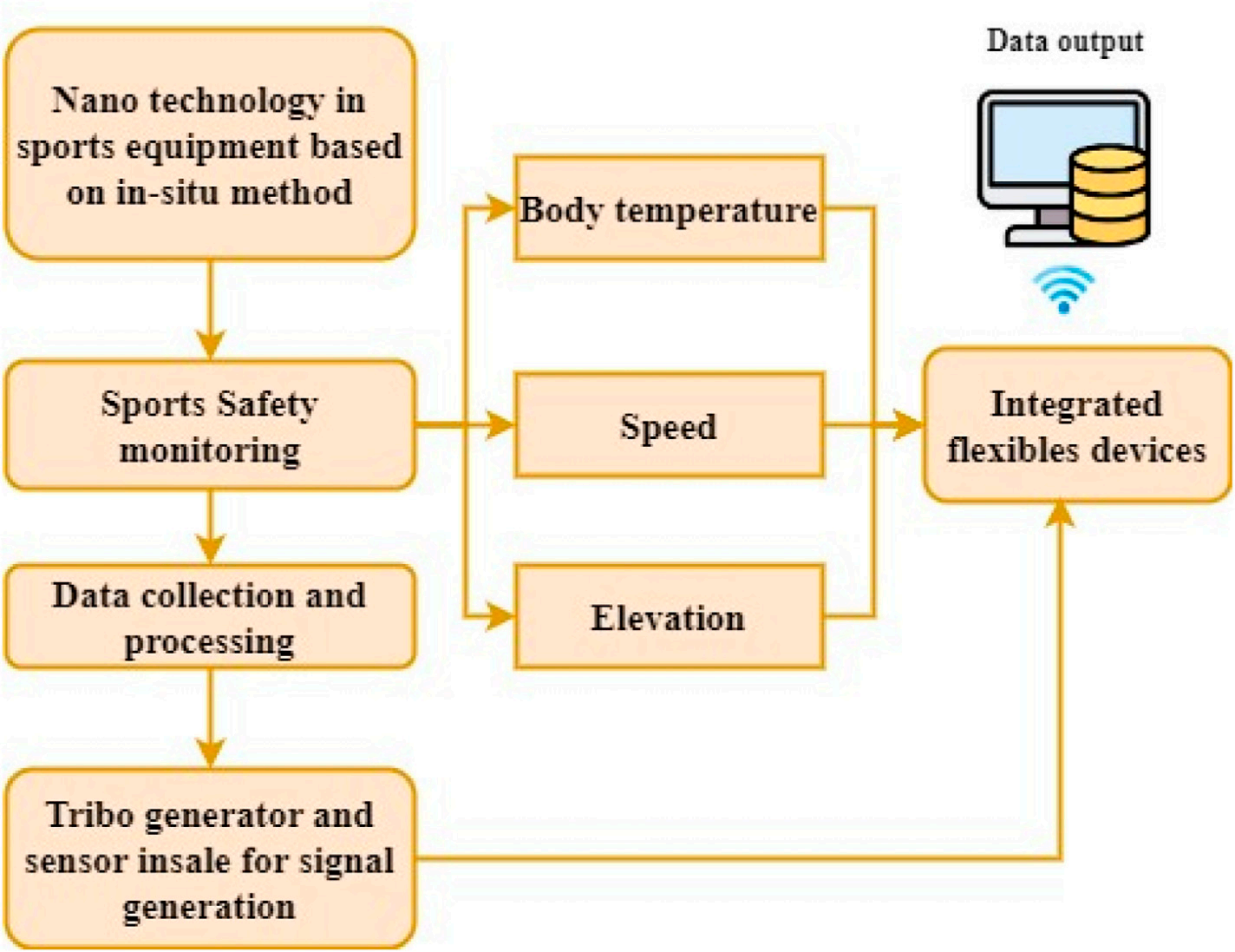
Nanotechnology-based sports equipment with flexible conjugated materials.

High-performance textiles have been increasingly developed and used in athletics and outdoor sports gear. Figure 2 illustrates the equipment for monitoring provided for the athlete in the sports event based on the nanotechnology and conjugate material. The performance requirements for many of these goods call for a careful balancing act between drape, warmth, moisture barrier, and physiological comfort. As a result of innovations in manufacturing and using suitable membranes made of polymers and surface finishes, it is now feasible to effectively marry the customer’s demands for aesthetics, design, and functionality in sportswear for various end-use applications. The essential components in providing the necessary physiological comfort level are suitable moisture and heat control based on nanotechnology, including Nanofibers, Nanocomposite fibers, and Nano-finishing. Unique qualities are added to the product manufacturing finishing techniques of highly designed textile-based sports products to attain the highest comfort and performance level in *in situ* Nano finishing. *In-situ* nanostructures have recently been acknowledged as a practical alternative to the traditional *ex-situ* method of finishing athlete equipment, which requires much time and effort. The *in situ* nanostructure finishing approach eliminates the requirement for an additional fixing step by creating nanoparticles in the structure of the textile material. For example, the mechanical properties of textiles can be improved thanks to the positive effects of the mechanical and biochemical attributes over time. To give fabric versatility like self-cleaning and antibacterial properties, Montazer and his research team have studied the *in situ* synthesis of various nanoparticles, such as silver, TiO2, ZnO, and TiO2/Ag nanocomposites, for the past 10 years. Metal and nanoparticles of copper oxide have been incorporated into various textiles, expanding their functional potential. The article explains how to make tribo Nano power production devices using flexible textile fabrics as frictional components. The report delves into how certain material qualities affect signal generation efficiency. There have been several breakthroughs in the study of triba Nanogenerators. Research efforts are being concentrated on selecting more malleable and less ephemeral materials. Almost all of the wearable textile fiber material criteria have been met.

The article investigates the potential of several fabric fiber types for the fabrication and use of tribo Nanogenerators, building on prior work with polymer tribo nanotechnology to achieve the ideal union of nanotechnology with textiles. In this paper, two types of fiber materials with substantial gain and loss electron pairs were chosen experimentally to create flexible triboelectric Nano power devices using the concept of tribo signal generation and electromagnetic induction. It has been integrated into the insoles at last. It harnesses the athlete’s kinetic energy by turning it into electricity, which is then used to track their health and fitness and ensure their safety throughout sporting events. Nano socks fabricated from Nano textile materials serve as nanotechnology and make up the bulk of the model. The athlete’s motion is converted into power, and the nanosensor records essential data about the training session.

✓ The effect of varying thicknesses of friction nanoparticles (PTFE and nylon) on power production performance was investigated in this experiment. The chosen fabrics are all plain weave; however, various weave patterns may significantly impact the effectiveness with which signal is generated.
✓ This experiment only looked at the effect of spacer fabric and yarn density on the effectiveness of sensor signal generating energy in the weaving process. It can investigate how different upper- and lower-layer yarns affect the efficiency of electricity generation.
✓ This article solely examines the impacts of two nanomaterials on the electric power performance of tribo nanotechnology due to the constraints of the available experimental materials. It can be used to test how different nanomaterials affect the final signal.
✓ The insole is the only place where the manufactured power production gadget is employed in this text. It can also offer power to other electronics when used with them.

The data is delivered to the user via a mobile phone app or wearable to monitor sports safety. The mathematical terms, packaging weight, and organization of the fabric’s component strands and construction are all carefully considered in developing activewear and sportswear to facilitate the necessary humidity and heat dispersion at high metabolism rates.

#### 3.1.2 Nanoscale -FCM-SE in athletic event

**FIGURE 3:**
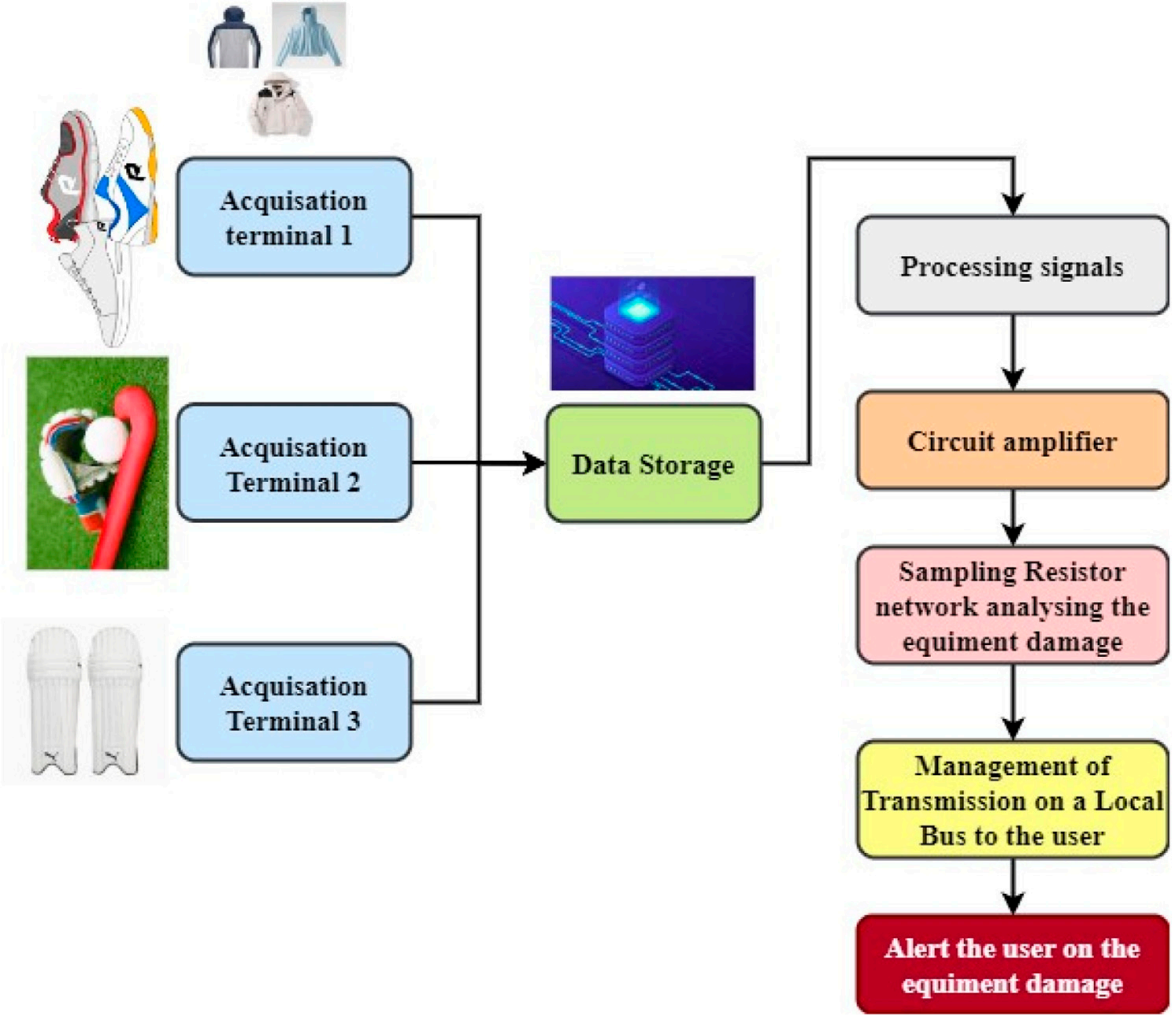
Flow structure of Nanoscale -FCM-SE in an athletic event.

In manufacturing sporting equipment, conjugated materials are typically used to provide additional layers of protection in Figure 2. The training procedure should include an adequate challenge and volume of physical activity. The academic level of athletic training and athletes’ performance can be improved through the analysis and decision-making of diverse training and competitive data. The information about database elements is culled from external sources via the training process’s data extraction procedure, integration, and transformation subsystem (Figure 3).

• The task of data acquisition is to import previously collected information into a database that processes the signals.
• The terminal sensors are connected to the equipment, and the information is in the database. Synthesis, reconstruction, screening, and transformation of the motion data are all possible because of the database’s structure, allowing for the creation of a single set of analytical data.
• Sensor technology encompasses the complicated technology behind the development and construction of sensors, which includes attention to detail and outstanding performance, and the technology behind using sensors in various contexts. Sensors can be broken down into three categories based on their intended use: physical, chemical, and biological.
• As a result, research into a technology to efficiently track training loads is essential, with sensor technology providing a means of doing so for athletes in various fields.
• As a result, better track athletes’ health and fine-tune their workouts to maximize their performance. It achieves optimum monitoring outcomes of the athletics condition by combining analysis findings of typical variables related to the load state of sports.
• Throughout a given time frame, athletes’ instruction, cardiovascular physical fitness, dietary habits, and performance at games are all things that can and should be monitored and controlled.

When subjected to external forces, minor, imperceptible shifts would occur in the connected material. The crystal’s structural strength shifts as the charge carriers focus on the motion. Because of this, there is a significant shift in its resistance value, and the resistor constructed from its parts experiences the same growth. The resistivity value of nano conjugate materials changes relatively under stress is represented in Equation (1)

$$\frac{∆P}{P}=\left(1+2\theta \right)\gamma +\frac{∆r}{r}$$ (1)

Where θ is the Nano-conjugate materials’ Poisson ratio, γ denotes the axial stress on the position of the equipment and $\frac{∆r}{r}$ is the resistivity.

The Nano conjugate material’s proportional resistance variation is shown in Eq. (2)

$$\frac{∆r}{r}=\delta Q\theta$$ (2)

Conjugated materials’ stress tolerance coefficient is given in Eq. (3)

$$S=\frac{∆P}{P}/\theta =\delta Q$$ (3)

Sensor-collected environmental signals typically have low amplitudes that necessitate amplification before they can be appropriately analyzed and interpreted. When combined with flexible sensors like pressure gauges and optical sensors, nanotechnology can be made from conjugate polymer components, one of the many applications of conjugated polymers for movement monitoring. It is connected to the skin and tracks anything from joint motion to skeletal muscle activity. They can follow and assess movements’ posture, intensity, and force in real-time by measuring pressure, stretching, and deformation. Nanomaterials can be integrated with conjugated materials to create intelligent textiles like brilliant athletic wear and bright socks with compression features. These garments can track the wearer’s vital signs and other real-time biometric data. Wireless connections allow them to deliver a pleasant wearable sensation and send data to portable devices for later study and recording. Optical sensors and their role in motion detection can be made with conjugated polymer materials.

After developing the state parameter designation of sports loads, the corresponding state motion monitoring identification is attained based on Eq. (4)

$$m\left(y\right)=\sum_{i=1}^{n}{x_{i}}^{*}c\left(y_{i}*y\right)+d^{*}$$ (4)

Here $d^{*}$ represents the statistic of the physical motion of the athlete, c is the load state feature recognition, and x & y indicates the focal points of the athlete’s activity.

During cardiac contraction workouts of athletes and the increased blood volume in the arteriovenous, the blood’s ability to absorb light fluctuates periodically when the heart is frequently beating because the amount of blood in the arteries fluctuates with each heartbeat. The sensor on the skin’s outer layer would use the reflected parabolic and diffuse radiation from an incident light source to determine the heart rate given in Equation (5)

$$H_{i}\left(t\right)=l\left(t\right)+y_{s}\left(t\right)+y_{d}\left(t\right)+y_{m}\left(t\right)$$ (5)

Where $l\left(t\right)$ is the light intensity level, $y_{s}\left(t\right)\&y_{d}\left(t\right)$ denotes spectral and diffusion components and $y_{m}\left(t\right)$ denotes the heart dilation level in motion.

When a person moves, the distance between them, the source of illumination, and the sensor shifts, altering the reflected factor. The strength and specifics of indicators vary in every guidance, and the process by which signals are generated would cause the human body to perceive the leader. Here, the precise expression of the signal flow is shown in Eq. (6)

$$\sum h_{i}\left(t\right)=-h_{r}+h_{c}+h_{b1}-h_{b2}+h_{b}+h_{q}$$ (6)

Here $h_{r}$ push force created by expulsion, $h_{c}$ represents the volume of blood pumped out with each heartbeat, $h_{b1}\&h_{b2}$ denotes the blood flow acceleration levels, $h_{b}$ is the blood flow in motion, and $h_{q}$ denotes signal strength in all directions.

Compared to the initial breathing signal, there is a noticeable average deviation. Clear pulmonary signs can be obtained by de-baselining the initial movement. The breathing signal of the thermal sensor results obtained is represented in Equation (7)

$$b\left(t\right)=\sum_{i=0}^{n}x_{i}t^{i}$$ (7)

Where n is the positive integer represents the order of the polynomial, $x_{i}$ is the frequency of the breathing signal, and t is the baseline drift.

Next, the sampling resistor will analyze the data of any changes or injuries in the existing event, and competition data for each team or event is stored in the system, along with historical data from previously used applications. Then, the information or alert is given to the coach and the athlete regarding the error. For the most part, athletes wear protective gear to safeguard their health and prevent injuries while competing. It includes clothing and pads constructed from conjugated materials.

The coach or athlete can modify their regimen based on the data collected. Quantity, quality, frequency, and duration of training are all part of this concept. Keeping an eye on these metrics is a great way to see if your training regimen is realistic and producing results. Supporting athletic endeavors and healthcare requires understanding how the characteristics of an intense situation regarding exercise might shed light on the present condition of treatment and administration of training.

#### 3.1.3 Rules and policies

Helmet guards, apparel, leg security personnel, security personnel, thin belts, and mouth guards are all permitted in national and provincial competitions for traditional martial arts following the current regulations of the General Administration of Sports of the People’s Republic of China. None of the competitors can utilize these essential protective gears, and all of them have to clear a pre-competition security check and be sealed before they can be used throughout the contest. But if the current local competition scene is any indication, protective apparel and equipment are rarely utilized. A squad member worries that their safety equipment would hinder their effectiveness and that the gear will be useless in protecting them beyond a certain point of violence. At the same time, athletes will need safety equipment to prevent bodily harm during competition and regular training.

There needs to be a greater emphasis on safety training and self-defense awareness for athletes. Because sports injuries are so common, athletes must have a foundational understanding of them. Students need to recognize different sports injuries, their severity, and their origins. It is also important to instruct children on how to take personal responsibility for preventing sports injuries by learning a variety of self-help measures. Students can better understand the dangers of athletic injuries and the significance of taking precautions to avoid them if they are introduced to these concepts through concrete examples.

## 4 Experimental results

The proposed model utilizes the NFL-QB-Shoulder-Injuries to examine the potential of nanoparticles paired with flexible sports conjugate materials for injury prevention in athletes. https://www.kaggle.com/datasets/georgedurrant/nfl-qb-shoulder-injuries (Kaggle, 2023). Regarding the upper limbs, shoulder injuries are consistently high on the list for both impact and noncontact sports. One-third of all athletic injuries can be attributed to them (Enger), significantly contributing to athlete morbidity. These factors highlight the significance of studying shoulder injuries and their subsequent healing parameters within orthopedics. Athletes are particularly vulnerable to shoulder injuries, often due to a direct blow or a fall onto the ipsilateral shoulder. Lateral and anterior glen humeral unpredictability, acromioclavicular pathology, and rotator cuff tears are some of the most common injuries in this group (Gibbs). The risk of shoulder injury, ranging from mild sprains to career-ending rips, is relatively high in American football due to the intense contact and high speeds at which the game is played. Almost half of NFL players have had a shoulder injury in the past, and 34% required surgery. Because of the nature of their profession and the connection between their throwing mechanics and other on-field actions, quarterbacks are especially vulnerable to shoulder problems. This research aimed to examine the effects of shoulder injuries on (1) general performance metrics among NFL quarterbacks and (2) the effects of procedures performed to repair these injuries on career outcomes based on indicators like passer rating, rushing yards, and passing touchdowns. After surgery on their injured shoulder, a quarterback in the National Football League was predicted to 1) perform worse than they had before the injury. Two) Surgical patients will outperform nonsurgical patients on all measures of performance. The performance metrics of the proposed model involve the 100 players using the equipment, including the degree of damage, Nano-FCM-SE Equipment Effectiveness, and Root Mean Square Error rate analysis of Nano-FCM-SE.

The 1980–2019 NFL Injured Reserve (IR) lists were queried from Pro Sports Transactions to discover quarterbacks with shoulder injuries. Some 50 quarterbacks with long-term shoulder issues had their dominant throwing arm surgery for the first time. For injury type and surgery dates, manual searches were done.

### 4.1 Degree of Damage

**FIGURE 4:**
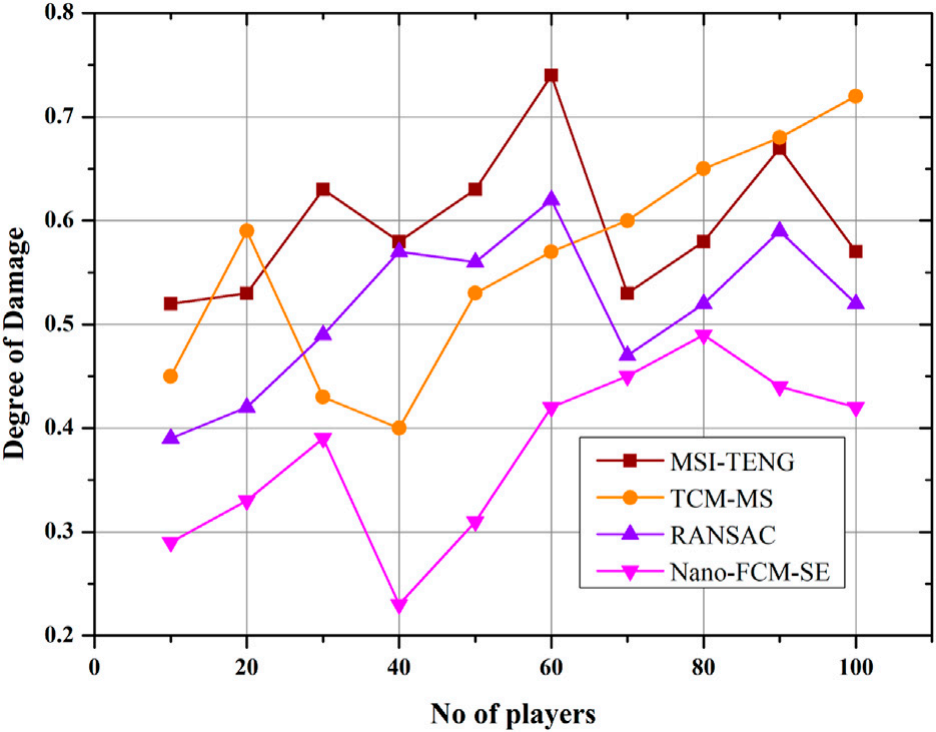
Degree of damage.

Degree of damage refers to the severity of the athlete’s injury during the sports event due to the failure of equipment, lack of training, and ignorance of rules and regulations, as represented in Figure 4. Increasing the sports training intensity has a negligible effect on the number of iterations but a significant effect on the amount of time spent computing. The above goal function of new Nano conjugate materials was tested in a structural damage identification verification study, in which damage models of varying intensities were chosen to use the velocity of motion as input, the calculated damage levels were used as measurement information, the model’s pre-damage state was known in full detail, and the objective functions were modified using a variety of approaches. Compared to the MSI-TENG, TCM-MS, and RANSAC algorithms, the proposed model causes significantly less harm.

### 4.2 Nano-FCM-SE Equipment Effectiveness

**FIGURE 5:**
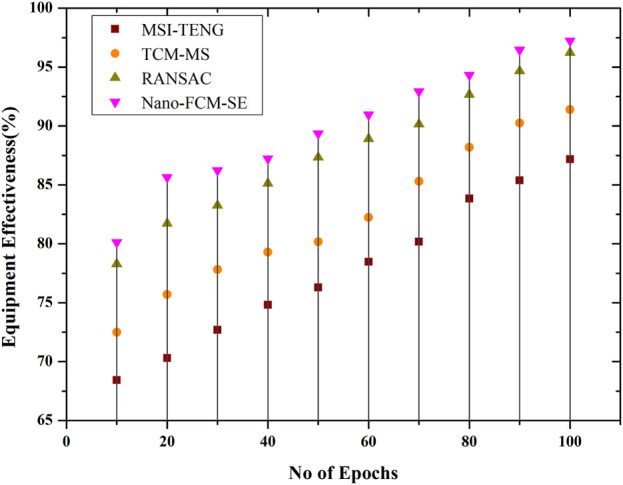
Nano-FCM-SE equipment effectiveness.

Sports need a considerable quantity of exertion and have complex posture requirements. Professional athletes are prone to injury for the reasons listed above. Due to the athlete’s temporary inability to recuperate from sport and training, muscle stiffness is a common post-injury problem during healing. Next, the Nano-FCM-SE data is analyzed, and it is discovered that using Nanotechnology and conjugate materials on apparatus and sensor technology could actively encourage the long-term promotion of protection and recovery of harm. In conclusion, the therapeutic applications of nanoparticles are outlined, and the multiple-stage comprehensive assessment model is employed to assess the effect of numerous regularly used biomaterials in providing damage and adhesion protection. The performance, adaptability, durability, and portability of sports equipment made with nanomaterial are all improved. The equipment shields the athlete from harm and affects their efficiency. Figure 5 shows the effectiveness of the proposed models on equipment is high compared to the other traditional models.

### 4.3 Athlete injury protection efficiency

**FIGURE 6:**
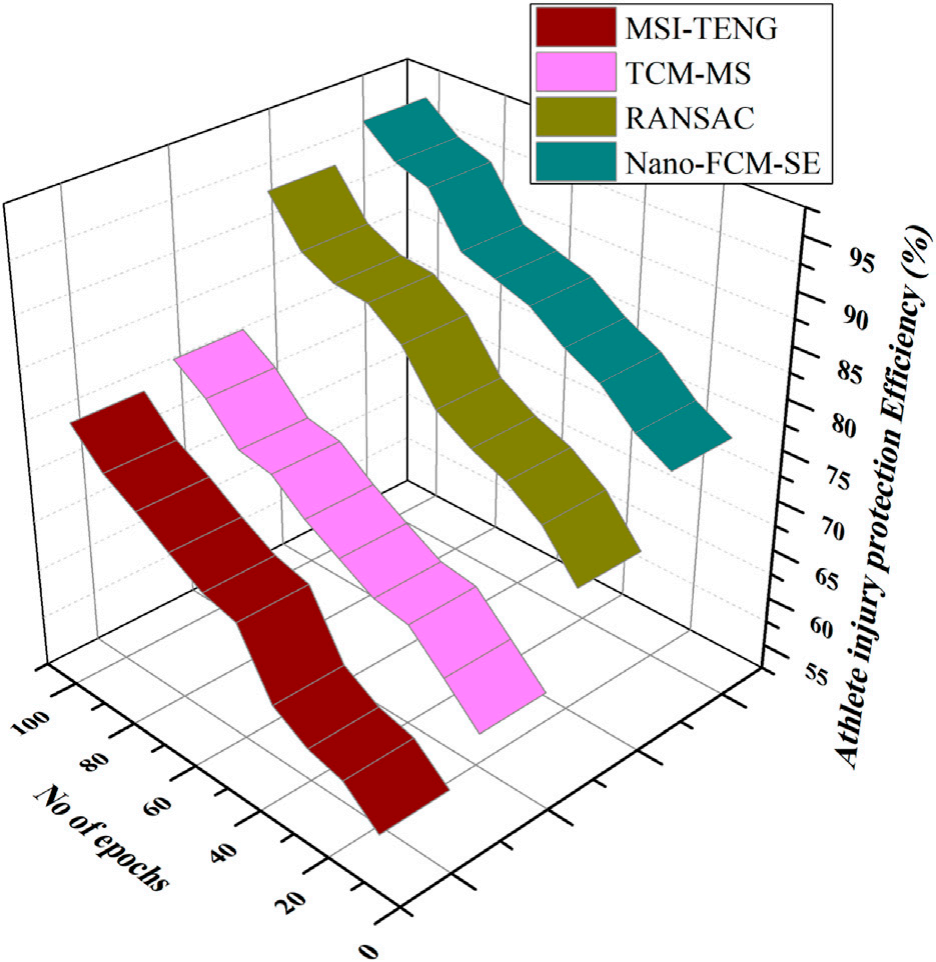
Athlete injury protection efficiency.

Figure shows the effectiveness of the of the parts of athletes sports injuries, it was realized that the injury rate of the pupil ankle joint was the highest; in the research of the types of athletic endeavours injuries among students, it was concluded that students were more likely to suffer from joint sprains; in the project investigation of students’ sports injuries, it was concluded that students were more prone to sports injuries in ball games with a large amount of exercise; in the investigation of the causes of students’ sports injuries, it was concluded that the main reasons for pupil athletic injuries were physical insufficiency, equipment and a detrimental location surroundings; in terms of the accomplishment assessment of the athletics injury protection system, it was discovered that the precision, performance, genuineness, and productivity of the sports injury automatic protection system based on Nano technology combined with the conjugate material on the equipment’s provided for the athletes technological advances had been improved to different degrees compared with the conventional sports injury protection methods. Compared to the *status quo*, the detection effectiveness of the sports-related injury protection system proposed in this study is 5.17 percentage points higher (Figure 6).

### 4.4 Root mean square error rate analysis of nano-FCM-SE

**FIGURE 7:**
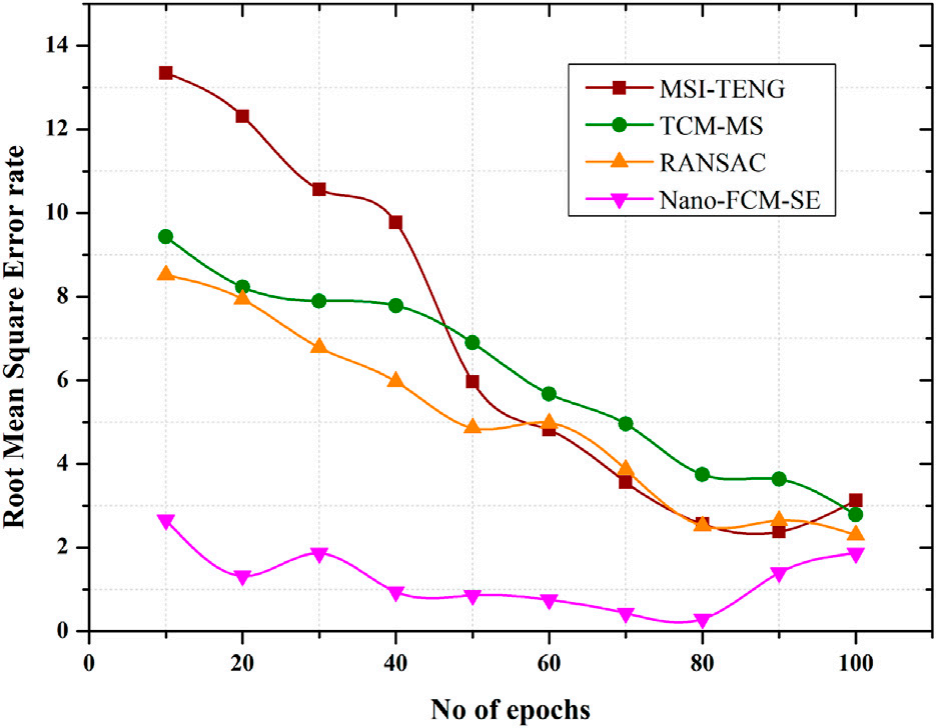
Root mean square error rate analysis of Nano-FCM-SE.

The Nano-FCM-SE technique’s root mean square error rate analysis is shown in Figure 7. The least amount of error and distortion in sensors is achieved when a hierarchical approach to protecting athletes from harm obtains the best RMSE value. The average degree of error in estimates is quantified by the root-mean-squared error (RMSE). It demonstrates how well the demonized thermal images match the measured temperatures. More accurate temperature readings can be obtained with this method since the RMSE values are reduced, indicating that the crucial thermal information has been preserved despite the reduced impact of nanotechnology. It is essential in sports medicine, where accurate temperature readings are required to assess athletes’ wellbeing and performance. The low RMSE value produced in an investigation of athletes’ mental health using the Nano-FCM-SE model demonstrates the model’s exceptional accuracy in forecasting mental wellbeing from sensor data. Disparities between forecasts and observations in terms of both mental health indicators and physical health are assessed using root-mean-squared error. The Nano-FCM-SE model effectively predicts athletes’ emotions due to its capacity to safeguard the individual’s complex elevation and interactions. Accurate and timely assessment of mental health in athletes is crucial for making appropriate therapeutic or training adjustments. In this context, RMSE values indicate a technique’s stability and dependability. This means Nano-FCM-SE that the model can accurately predict athletes’ psychological wellbeing using thermal data and that the hierarchical thermal noise reduction strategy has dramatically improved the thermal picture quality. These results highlight the importance of precise data for athlete safety and performance, highlighting the utility of these approaches in athlete protection on Nano conjugate materials and sports science on sporting events and equipment.

The proposed approach produces far less adverse outcomes than algorithms like MSI-TENG, TCM-MS, and RANSAC. Compared to competing models, the proposed one has poor performance metrics regarding damage severity (0.2). Nanomaterial-made sporting goods are superior in every way: efficiency, versatility, longevity, and portability. Their gear impacts the athlete’s safety and performance. Compared to the other conventional models, the presented models exhibit a high level of efficacy on equipment (Figure 5). The detection efficiency of the sports injury protection system described in this research is 5.17 percentage points higher than the current state of the art. A hierarchical strategy yields the best root-mean-squared error (RMSE) number when keeping athletes safe. The findings demonstrate the value of such techniques in athlete safety on Nano conjugate materials and sports biology on sporting events and equipment, emphasizing the significance of accurate data for athlete safety and performance.

Nano-conjugated sporting gear materials are examined for water resistance, flexibility, low weight, visual appeal, ventilation, and longevity. Flexible conjugated materials in athletic training are showing promise for improving performance and growing sports. In the end, scientific training helps athletes and coaches reduce sports injuries; thus, athletic sports professionals and employees should prioritize player training. Therefore, effective training methods and academic athlete medical aid can ensure athlete health and training quality in future investigations.

The article examines the impact of two nanomaterials on the sensor output performance of tribo nanogenerators, as this is all that could be tested in the laboratory. It can be used to test how different nanomaterials affect the final signal.

## 5 Discussion

The fluctuating preponderance of sports-related injuries and irregular rehabilitation center distribution in India is concerning. Sports difficulties include misinterpreting players’ body language, wearing uncomfortable clothes, and disliking equipment. This study investigates whether nanoparticles and sports-specific flexible conjugate materials can reduce injury risk. Nanotechnology mixed with flexible conjugated materials in sports events (Nano-FCM-SE) with excellent optical, electrical, and other qualities can prevent injuries. The review covers nanomaterials in sports training. It examines how conjugated materials can improve athlete training, sports monitoring, and equipment. Nano-conjugated sporting gear materials are analyzed for water resistance, adaptability, low weight, attractiveness, breathability, and durability. Flexible conjugated materials in athletic training are showing promise for improving performance and growing sports.

In conclusion, scientific training helps athletes and coaches reduce sports injuries; thus, athletic sports professionals and workers should prioritize player training. The proposed method yields less negative results than MSI-TENG, TCM-MS, and RANSAC. The proposed damage severity model performs poorly relative to competitors (0.2). Nanomaterial-made sports gear is more efficient, versatile, durable, and portable. Gear affects athletes’ safety and performance. Compared to conventional models, the given models are effective on equipment (Figure 5). The sports injury protection system reported in this research has 5.17 percentage points greater detection efficiency than the current state of the art. Hierarchical strategies get the best root-mean-squared error (RMSE) for athlete safety. The findings of such methodologies in athlete safety on Nano conjugate materials and sports biology on sporting events and equipment underline the importance of precise data for athlete safety and performance.

## 6 Conclusion

Athletes are vulnerable to various injuries due to their high-intensity training. However, a significant issue is the prevalence of sports-related injuries, especially among young people. Sports injuries directly and indirectly impact the regular education and lives of college graduates. Therefore, boosting student athletes’ performance and encouraging healthy growth requires an awareness of the prevalence and fundamental causes of injury among college athletes. The fluctuating majority of sports-related injuries and the inconsistent distribution of rehabilitation centers throughout India are worrying trends. Several potential problems might occur in sports, including incorrectly interpreting players’ bodily signs, wearing uncomfortable attire, and being unhappy with sports equipment. This research looks into the feasibility of using nanoparticles in conjunction with sports-specific flexible conjugate materials to reduce the risk of injury. It is proposed that Nanotechnology be combined with flexible conjugated materials in sports events (Nano-FCM-SE) with outstanding visual, electronic, and other properties to sports products to prevent injury. The review summarizes the uses of Nanomaterials in sports training. It investigates the potential of conjugated materials in improving training effects for athletes, monitoring the status of sports, and enhancing equipment. Nano-conjugated materials for athletic gear are tested for various desirable qualities, including but not limited to water resistance, adaptability, low weight, attractiveness, breathability, and durability. There is mounting evidence that incorporating flexible conjugated materials into athletic training can boost athlete performance and contribute to the growth of the sporting world. Only when paired with the insole is the produced nano equipment gadget discussed in this article effective. It can also be used with other sensor energy devices based on conjugate materials. In conclusion, scientific training is an essential approach for athletes and coaches to decrease sports injuries; thus, athletic sports professionals and workers should pay sufficient consideration to the players’ training. Therefore, in future studies, the quality of training and the health of athletes can be double guaranteed by appropriate training techniques and scientific, athletic medical assistance.

Due to the complexity of the device and the high computational cost, laboratory testing was limited; thus, this paper only looks at the effect of two nanomaterials on the sensor output performance of tribo nanogenerators. It can be used to investigate the effects of various nanomaterials on the transmitted signal.

## Data Availability

The original contributions presented in the study are included in the article/supplementary material, further inquiries can be directed to the corresponding authors.

## References

[B1] Alheib O. da Silva L. P. da Silva Morais A. Mesquita K. A. Pirraco R. P. Reis R. L. (2022). Injectable laminin-biofunctionalized gellan gum hydrogels loaded with myoblasts for skeletal muscle regeneration. Acta Biomater. 143, 282–294. 10.1016/j.actbio.2022.03.008 35278687

[B2] Ansari A. A. (2022). “Nanotechnological advancements in sports rehabilitation to elevate athletic performance levels,” in International conference on nanotechnology: opportunities and challenges (Singapore: Springer Nature Singapore), 377–381.

[B3] Cai Y. Pan Y. Liu L. Zhang T. Liang C. Mou X. (2023). Succinct croconic acid-based near-infrared functional materials for biomedical applications. Coord. Chem. Rev. 474, 214865. 10.1016/j.ccr.2022.214865

[B4] Cao X. Xia J. Meng X. Xu J. Liu Q. Wang Z. (2019). Stimuli-Responsive DNA-gated nanoscale porous carbon derived from ZIF-8. Adv. Funct. Mat. 29, 1902237. 10.1002/adfm.201902237

[B5] Chen-Yu Z. Qian-Jin L. Juan-Juan H. Yu-Ting S. Qing-Yi Z. Nie R. (2022). Design of biopolymer-based hemostatic material: starting from molecular structures and forms. Mater. Today Bio 17, 100468. 10.1016/j.mtbio.2022.100468 PMC9626749 36340592

[B6] Chouhan S. Butola B. S. (2023). “Exploration of UV absorbing functional materials and their advanced applications,” in Functional materials from carbon, inorganic, and organic sources (Sawston, United Kingdom: Woodhead Publishing), 187–243.

[B7] Fan J. Abedi-Dorcheh K. Sadat Vaziri A. Kazemi-Aghdam F. Rafieyan S. Sohrabinejad M. (2022). A review of recent advances in natural polymer-based scaffolds for musculoskeletal tissue engineering. Polymers 14 (10), 2097. 10.3390/polym14102097 35631979 PMC9145843

[B8] Gao D. Liu J. Lou J. Tian S. J. Lian H. X. Niu S. M. (2022). Medical services for sports injuries and illnesses in the Beijing 2022 Olympic Winter Games. World J. Emerg. Med. 13 (6), 459. 10.5847/wjem.j.1920-8642.2022.106 36636567 PMC9807383

[B9] Han F. Wang T. Liu G. Liu H. Xie X. Wei Z. (2022). Materials with tunable optical properties for wearable epidermal sensing in health monitoring. Adv. Mater. 34 (26), 2109055. 10.1002/adma.202109055 35258117

[B10] Hassabo A. G. Elmorsy H. Gamal N. Sediek A. Saad F. Hegazy B. M. (2023). Applications of nanotechnology in the creation of smart sportswear for enhanced sports performance: efficiency and comfort. J. Text. Coloration Polym. Sci. 20 (1), 11–28.

[B11] He Y. Kukhta N. A. Marks A. Luscombe C. K. (2022). The effect of side chain engineering on conjugated polymers in organic electrochemical transistors for bioelectronic applications. J. Mater. Chem. C 10 (7), 2314–2332. 10.1039/d1tc05229b PMC8852261 35310858

[B12] Hjort K. Vicini I. Mårtensson G. (2023). “Smart bioelectronic and wearable systems: SINTEC final workshop: proceedings,” in SINTEC Final Workshop "Smart Bioelectronic and Wearable Systems, Uppsala, April 26-28, 2023.

[B13] Javed N. Pottoo F. H. Barreto G. E. (2022). Nano-nutraceuticals dietary supplements for athletes: an eminent approach for managing traumatic brain injuries. Curr. Mol. Pharmacol. 15 (1), 1–2. 10.2174/1874467215666220104212247 35048803

[B14] Kaggle (2023). NFL-QB-Shoulder-Injuries. Available at: https://www.kaggle.com/datasets/georgedurrant/nfl-qb-shoulder-injuries

[B15] Li G. (2022). Machine leaning-based optimization algorithm for myocardial injury under high-intensity exercise in track and field athletes. Comput. Intell. Neurosci. 2022, 1–9. 10.1155/2022/7792958 PMC9110131 35586102

[B16] Li J. Jiang Y. Dai Q. Yu Y. Lv X. Zhang Y. (2022). Protective effects of mefunidone on ischemia-reperfusion injury/Folic acid-induced acute kidney injury. Front. Pharmacol. 13, 1043945. 10.3389/fphar.2022.1043945 36506525 PMC9727196

[B17] Lv M. Cao X. Tian M. Jiang R. Gao C. Xia J. (2022). A novel electrochemical biosensor based on MIL-101-NH2 (Cr) combining target-responsive releasing and self-catalysis strategy for p53 detection. Biosens. Bioelectron. 214, 114518. 10.1016/j.bios.2022.114518 35780541

[B18] Miller M. R. Robinson M. Fischer L. DiBattista A. Patel M. A. Daley M. (2021). Putative concussion biomarkers identified in adolescent male athletes using targeted plasma proteomics. Front. neurology 12, 787480. 10.3389/fneur.2021.787480 PMC8721148 34987469

[B19] Ramos D. Almeida L. (2023). Managing nanomaterials in the workplace by using the control banding approach. Int. J. Environ. Res. Public Health 20 (11), 6011. 10.3390/ijerph20116011 37297615 PMC10252431

[B20] Rao K. M. Kim E. Kim H. J. Uthappa U. T. Han S. S. (2023). Hyaluronic acid-quercetin pendant drug conjugate for wound healing applications. Int. J. Biol. Macromol. 240, 124336. 10.1016/j.ijbiomac.2023.124336 37030466

[B21] Robert G. (2021). A win-win situation between sports and natural environment protection based on the theory of cooperation and competition. Nat. Environ. Prot. 2 (3), 50–58. 10.38007/NEP.2021.020306

[B22] Rodriguez-Santiago B. Castillo B. Baerga-Varela L. Micheo W. F. (2019). Rehabilitation management of rotator cuff injuries in the master athlete. Curr. sports Med. Rep. 18 (9), 330–337. 10.1249/jsr.0000000000000628 31503045

[B23] Tan Y. Cai B. Li X. Wang X. (2023). Preparation and application of biomass-based sprayable hydrogels. Pap. Biomaterials 8 (2), 1–19. 10.26599/pbm.2023.9260006

[B24] Wang D. (2022). Analysis and research on regeneration therapy of athlete tendon injury based on nanometre sensor technology. Int. J. Nanotechnol. 19 (6-11), 861–873. 10.1504/ijnt.2022.10054008

[B25] Willwacher S. Bruder A. Robbin J. Kruppa J. Mai P. (2023). A multidimensional assessment of a novel adaptive versus traditional passive ankle sprain protection systems. Am. J. Sports Med. 51 (3), 715–722. 10.1177/03635465221146294 36734465 PMC9983046

[B26] Younas A. Gu H. Zhao Y. Zhang N. (2021). Novel approaches of the nanotechnology-based drug delivery systems for knee joint injuries: a review. Int. J. Pharm. 608, 121051. 10.1016/j.ijpharm.2021.121051 34454029

[B27] Zhang N. (2022a). Promoting outdoor sports tourism by natural resources and environment protection. Nat. Environ. Prot. 3 (Issue 1), 44–51. 10.38007/NEP.2022.030106

[B28] Zhang R. (2022b). Application of biomaterials in tendon injury healing and adhesion in sports. J. Healthc. Eng. 2022, 1–9. 10.1155/2022/5087468 PMC9018196 35449831

[B29] Zhou W. Chu H. (2022). Identification of sports athletes' high-strength sports injuries based on NMR. Scanning 2022, 1–7. 10.1155/2022/1016628 PMC9307404 35912121

[B30] Zhu Y. Zhao T. Sun F. Jia C. Ye H. Jiang Y. (2023). Multi-functional triboelectric nanogenerators on printed circuit board for Metaverse sport interactive system. Nano Energy 113, 108520. 10.1016/j.nanoen.2023.108520

